# Cellular and molecular response of dental stem cells to decellularized extracellular matrix scaffolds in regenerative endodontics: a systematic review

**DOI:** 10.3389/fdmed.2026.1766825

**Published:** 2026-03-06

**Authors:** Hanna Saith, Shishir Shetty, Nireeksha

**Affiliations:** Nitte (Deemed to be University), AB Shetty Memorial Institute of Dental Sciences (ABSMIDS), Department of Conservative Dentistry and Endodontics, Mangalore, India

**Keywords:** angiogenesis, biomaterials, decellularized extracellular matrix (dECM), dental pulp regeneration, dental pulp stem cells (DPSCs), ECM hydrogel, extracellular matrix scaffold, odontogenic differentiation

## Abstract

**Background:**

Decellularized extracellular matrices (dECMs) have gained increasing attention in regenerative dentistry due to their ability to replicate aspects of the native cellular microenvironment while reducing immunogenicity. Dental-derived stem cells exhibit regenerative and immunomodulatory properties, making them promising candidates for tissue repair when combined with biologically derived scaffolds such as dECMs.

**Objective:**

This systematic review aimed to evaluate the cellular and molecular responses of dental-derived stem cells exposed to decellularized extracellular matrix scaffolds in experimental *in vitro* models.

**Methods:**

A comprehensive literature search was conducted across PubMed, Scopus, and Web of Science to identify relevant *in vitro* studies investigating interactions between dental stem cells and decellularized extracellular matrix scaffolds. Ten studies published between 2015 and 2024 met the eligibility criteria and were included. Data on cell viability, adhesion, proliferation, migration, differentiation, and gene or protein expression were extracted. Due to heterogeneity in stem cell sources, scaffold origins, and decellularization protocols, a qualitative synthesis was performed. Risk of bias was assessed using the Quality Assessment Tool for *In Vitro* studies tool.

**Results:**

Across the included studies, dECM scaffolds were generally associated with favorable cellular responses, including improved cell attachment, survival, and proliferation. Molecular analyses frequently reported increased expression of markers related to odontogenic and osteogenic differentiation, extracellular matrix remodeling, and cell–matrix interactions. However, the magnitude and consistency of these responses varied according to the dECM source and decellularization methodology. The majority of the studies demonstrated a medium risk of bias, reflecting limitations in methodological reporting and experimental design.

**Conclusion:**

The current *in vitro* evidence suggests that dECM scaffolds may support beneficial cellular and molecular responses in dental-derived stem cells. Nevertheless, confidence in these findings is constrained by methodological heterogeneity, the lack of standardized protocols, and a predominance of studies with medium risk of bias. Consequently, these findings should be interpreted as preliminary, underscoring the need for rigorously designed and standardized preclinical investigations prior to clinical translation to regenerative dental therapies.

**Systematic Review Registration:**

https://doi.org/10.17605/OSF.IO/Z25HR, identifier Z25HR.

## Introduction

The animal kingdom includes organisms such as planaria, axolotls, and starfish that exhibit remarkable regenerative capacity; however, humans possess only limited regenerative potential. In dentistry, conventional approaches such as root canal therapy have long been the standard of care, with the primary aim of eliminating infection and preserving tooth structure (1). Although clinically effective, these methods are fundamentally reparative rather than regenerative.

Advances in biomedical sciences have driven a shift toward regenerative endodontics, which integrates stem cells, scaffolds, and signaling molecules to restore dental tissue vitality and promote continued root development in immature teeth (2). The growing emphasis on regenerative dentistry reflects the expanding role of molecular biology and tissue engineering in developing biologically driven therapeutic strategies.

Regenerative endodontic therapies have also demonstrated the potential to restore tooth vitality, nociception, and defensive responses against microbial and noxious stimuli (3). Central to these approaches are mesenchymal stem cells, which are multipotent cells involved in tissue regeneration and capable of differentiating into odontoblast-like and other mesenchymal lineages, with dental tissues serving as an accessible source.

Dental-related MSC populations include dental pulp stem cells (DPSCs), dental follicle progenitor cells (DFPCs), stem cells from the apical papilla (SCAP), tooth germ stem cells (TGSCs), periodontal ligament stem cells (PDLSCs), and stem cells from human exfoliated deciduous teeth (SHED) (4). These cells exhibit fibroblast-like morphology, express MSC-specific surface antigens, and possess multilineage differentiation and immunomodulatory capabilities, making them attractive candidates for regenerative applications.

Regenerative therapies rely on three key components, namely, stem cells, signaling molecules, and scaffolds. Scaffolds provide a three-dimensional framework that mimics the native extracellular matrix, supporting cell adhesion, proliferation, differentiation, and tissue regeneration. Scaffolds are broadly classified into natural and synthetic types. Synthetic scaffolds, such as polylactic acid, polyglycolic acid, and hydroxyapatite composites, are widely used due to their controlled architecture, mechanical strength, and predictable degradation profiles. However, despite these advantages, synthetic scaffolds lack intrinsic biological cues required for stem cell differentiation, resulting in limited cellular, vascular, and functional responses (5, 6).

In contrast, biological scaffolds have demonstrated superior biological performance. Decellularized extracellular matrices (dECMs) have emerged as promising biological scaffolds due to their ability to preserve native extracellular architecture while eliminating cellular and genetic components. dECMs can be derived from various tissues, including human and animal dental pulp and granulomatous tissues, and provide biochemical and structural cues that support stem cell adhesion, proliferation, and lineage-specific differentiation (7–9).

Experimental studies have reported that dECMs enhance key stem cell functions, including migration, proliferation, and multilineage differentiation, as well as neurogenic and angiogenic responses essential for dentin–pulp complex regeneration (7, 8). Furthermore, dECMs are considered immunologically safe, with a low risk of scaffold rejection, and their close resemblance to the native cellular niche is thought to facilitate more effective regenerative processes. The preparation of dECMs involves decellularization protocols designed to remove cellular and immunogenic components while preserving extracellular matrix structure and bioactive molecules. These protocols commonly employ chemical detergents, enzymatic digestion, and physical methods such as freeze–thaw cycles (10, 11).

Although dental tissues are the most frequently used dECM sources, non-dental tissues such as small intestinal submucosa, bladder, and cardiac extracellular matrices have also been explored. While these non-dental sources lack tissue specificity, they provide broad regenerative cues. In addition, an extracellular matrix produced using cultured dental stem cells has been investigated as a biologically relevant scaffold enriched with mineralization-promoting factors (6, 8). Collectively, these findings highlight the versatility of dECMs as biological scaffolds and their potential to enhance stem cell responses more effectively than synthetic materials (12). Dental-derived dECMs, in particular, offer tissue-specific cues that are advantageous for dentin–pulp complex regeneration and remain widely used in regenerative endodontics (7, 13, 14).

The objective of this systematic review was to synthesize the available *in vitro* evidence evaluating the molecular and cellular responses of dental-derived stem cells to decellularized extracellular matrix scaffolds in the context of regenerative endodontics, using the PICO framework (Population: dental stem cells; Intervention: dECM scaffolds; Comparator: alternative scaffolds or no scaffold; Outcomes: cellular and molecular responses).

## Materials and methods

The content of this article is presented in accordance with the Preferred Reporting Items for Systematic Reviews and Meta-Analyses (PRISMA) 2020 guidelines. The study was registered in the OSF registries under the DOI number: doi: 10.17605/OSF.IO/Z25HR. A systematic search was conducted in PubMed, Scopus, and Web of Science, and it was limited to English literature, as detailed in Table 1.

**Table 1:** Search strategy.

PubMed	Scopus	Web of Science
(“decellularized extracellular matrix” OR “decellularised extracellular matrix” OR “extracellular matrix scaffold” OR “dental pulp extracellular matrix” OR “amniotic membrane extracellular matrix”) AND (“dental pulp” OR “regenerative endodontics” OR “pulp regeneration”)	TITLE-ABS-KEY(”decellularized extracellular matrix” OR “decellularised extracellular matrix” OR “extracellular matrix scaffold” OR “dental pulp extracellular matrix” OR “amniotic membrane extracellular matrix”) AND TITLE-ABS-KEY(”dental pulp” OR “regenerative endodontics” OR “pulp regeneration”)	TS = (“decellularized extracellular matrix” OR “decellularised extracellular matrix” OR “extracellular matrix scaffold” OR “dental pulp extracellular matrix” OR “amniotic membrane extracellular matrix”) AND TS = (“dental pulp” OR “regenerative endodontics” OR “pulp regeneration”)

Studies from 2015 to 2024 were targeted. The literature search was conducted with a combination of keywords, such as decellularized extracellular matrix, regenerative endodontics, and *in vitro*.

A backward search of citations was conducted to review the reference list of all the included studies.

The PICO question of this study is “Which cellular and molecular responses are found in dental-derived stem cells as a response to the use of decellularized extracellular matrices in regenerative endodontics?” (P = dental stem cells, I = decellularized extracellular matrix, C = alternative scaffold/no scaffold/conventional methods, O = cellular and molecular response).

The articles that satisfied the following inclusion criteria were added to the systematic review: articles published in English, *in vitro* study designs that evaluated dECM scaffolds, studies using dental-derived stem cells, and studies conducted from 2015 to 2024.

Non-English publications, *in vivo* studies, case reports, studies incorporating any adjuvants, studies using stem cells from cell lines other than DPSCs, and studies evaluating outcomes unrelated to regenerative endodontics were excluded.

**Figure 1:**
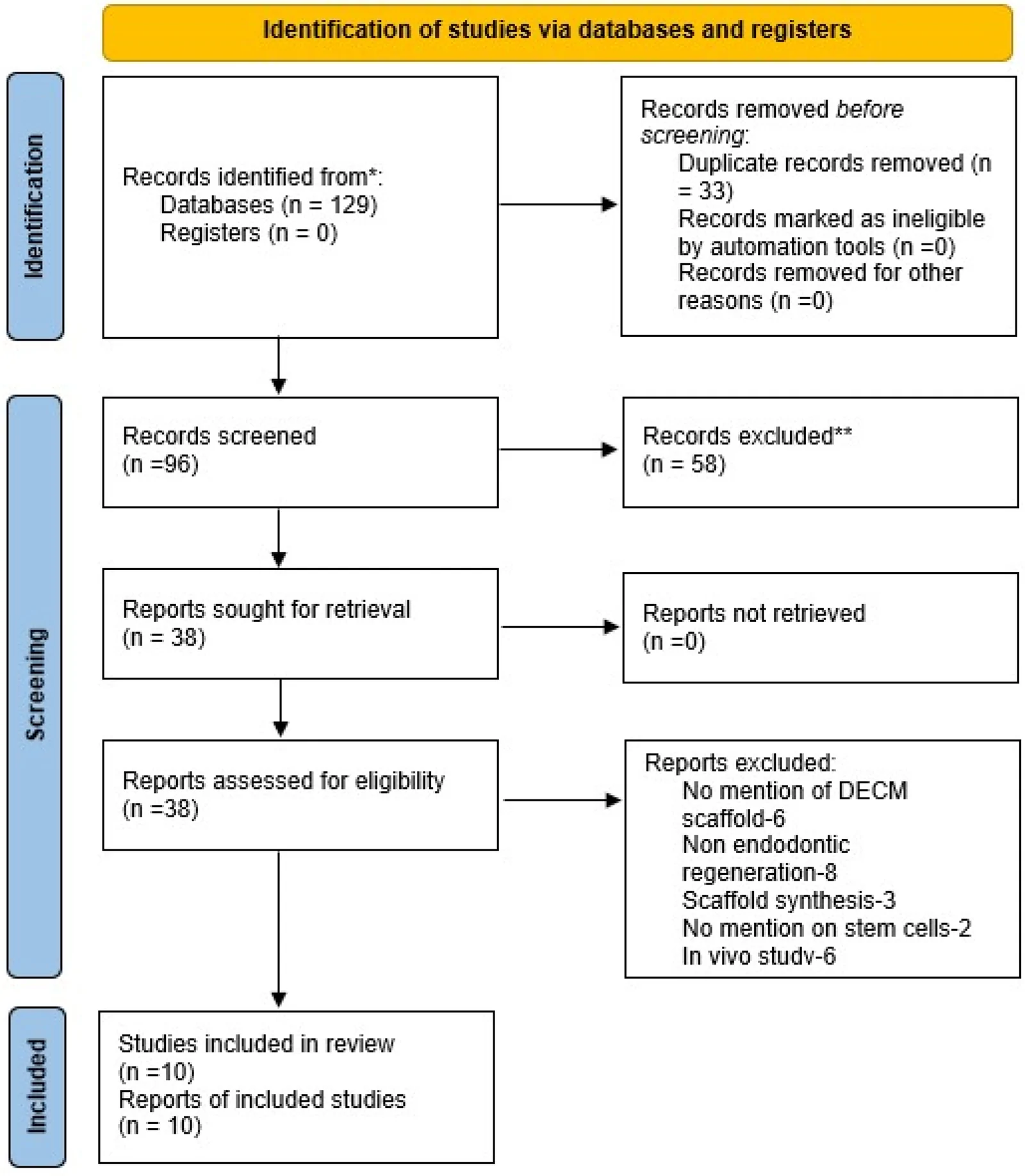
PRISMA 2020 flow diagram.

The titles and abstracts of selected papers were screened first, and further full-text screening was conducted to evaluate whether the studies were eligible under the predefined eligibility criteria, as illustrated in Figure 1.

### Risk of bias

The QUIN (Quality Assessment tool for *In Vitro* Studies) tool was used to evaluate the risk of bias in the included studies. In total, the following 12 methodological domains are assessed by the QUIN tool (Table 2): objective clarity, sample size justification, sampling technique, comparison group description, methodological detail, operator information, randomization, outcome measurement techniques, outcome assessor blinding, statistical analysis, results presentation, and disclosure of conflicts of interest.

**Table 2:** QUIN risk of bias.

Study	Clearly stated aims/objectives	Sample size calculation/justification	Sample source/selection	Randomization	Control groups	Methodology detail	Operator information	Blinding	Outcome measures	Statistical analysis	Data presentation	COI/ Fundi ng	Tot al (2 4)	Bias level
Bakhtiar et al. (2023) (19)	2	0	2	0	2	2	0	0	2	2	2	2	16	Medium
Bakhtiar et al. (2022) (23)	2	0	2	0	2	2	0	0	2	2	2	2	16	Medium
Elnawam et al. (2024) (18)	2	0	2	2	2	2	0	0	2	2	2	2	18	Low
Hu et al. (2017) (15)	2	0	2	0	2	2	0	0	2	2	2	2	16	Medium
Hu et al. (2024) (17) (PLdECM)	2	0	2	0	2	2	0	0	2	2	2	2	16	Medium
Matoug-Elwerfelli et al. (2018) (3)	2	0	2	0	2	2	0	0	2	2	2	2	16	Medium
Shi et al. (2023) (16)	2	0	2	0	2	2	0	0	2	2	2	2	16	Medium
Smith et al. (2015) (22)	2	0	2	NA	2	2	0	0	2	2	2	2	16	Medium
Song et al. (2017) (23)	2	0	2	0	2	2	0	0	2	2	2	2	16	Medium
Zhang et al. (2024) (20)	2	0	2	0	2	2	0	0	2	2	2	2	16	Medium

Each domain received a score of 0 (not reported/unclear), 1 (partially reported), or 2 (adequately stated). Based on the number of relevant domains, a total score and a percentage score were computed for each study. Research was divided into three categories: low risk (≥67%), medium risk (50%–66%), or high risk (<50%).

The QUIN risk-of-bias assessment was conducted independently by two reviewers. Disagreements were resolved through discussion and consensus. Studies were not eliminated only on the basis of the risk-of-bias score; instead, the final QUIN scores were utilized for qualitative interpretation and sensitivity analysis.

## Results

The literature search produced a total of 129 articles. After removing duplicates, 96 studies were screened, after which 38 articles underwent full-text screening. Finally, 10 articles were found eligible for the review.

A customized data extraction template was employed to extract relevant information from all the eligible studies, utilizing the following predefined headings.

### Risk of bias

Risk-of-bias assessment using the QUIN tool predominantly demonstrated medium methodological quality among the included studies. Nine of the 10 studies (90%) were classified as medium risk, while one study (10%) demonstrated a low risk of bias. No studies were rated as high risk. The most frequent deficiencies were the absence of sample size justification, lack of operator information, absence of blinding, and limited reporting of randomization procedures. Conversely, aims, methodological descriptions, data presentation, and statistical analyses were generally well reported. These findings indicate that although the *in vitro* evidence base is methodologically acceptable, the overall confidence in the findings remains moderate.

## Discussion

Evidence from multiple *in vitro* investigations evaluating scaffolds based on dECMs obtained from human, bovine, swine, periapical lesions, amniotic membrane, and rat submandibular gland tissues was gathered for this systematic review as in Table 3. Consistently high biocompatibility and strong support for stem cell adhesion, viability, proliferation, migration, and odontogenic differentiation throughout the experiments further reinforced dECMs' potential in regenerative endodontics. These responses align with the biological role of native ECMs, which provide biochemical, structural, and mechanical cues essential for tissue-specific lineage commitment. Collectively, these findings suggest that dECM scaffolds may mimic aspects of the natural pulp niche and therefore hold translational relevance in regenerative endodontics.

**Table 3:** Data extraction.

Author/year	Title	Scaffold source	Decellularization protocol	Stem cell type	Cellular and molecular outcomes
Hu et al. (2017) (15)	Decellularized swine dental pulp as a bioscaffold for pulp regeneration	Swine dental pulp	10% SDS + Triton X-100 (SDS rocked for 32 h at 25 °C; Triton X-100 for 2 h) +DNAase/ RNAase + sterilization + extensive washing to remove detergents	DPSCs	Adhesion, proliferation, and differentiation increased significantly.
Matoug-Elwerfelli et al. (2018) (3)	A biocompatible decellularized pulp scaffold for regenerative endodontics	Human dental pulp	Wishaw-based mild protocol: freeze thaw+0.03%SDS (24 h) in hypotonic buffer + nuclease treatment, peractic acid disinfection	DPSCs	Good cell viability
Shi et al. (2023) (16)	Decellularized rat submandibular gland as an alternative scaffold for dental pulp regeneration	Rat submandibular gland	SDS (10% w/v in deionized water for 32 h changed every 8 h followed by 1% Triton ×100 2 h, DNAase and RNAase	HDPSC RDPSCs	Significant Adhesion, proliferation, and odontogenic marker expression
Hu et al. (2024) (17)	Periapical lesion-derived decellularized extracellular matrix as a potential solution for regenerative endodontics	Periapical lesions (from patients aged 18 to 45)	0.2%SDS (12 h, 4 °C)*2+ DNase(50 micro/mL, 1 hr, 37 °C)	PLDSCs	Good proliferation, odontogenic and angiogenic expression (VEGF, TGF Beta, FGF2, and OPN)
Zhang et al. (2024) (20)	Decellularized human amniotic membrane scaffolds: influence on the biological behavior of dental pulp stem cells	Human amniotic membrane	Seven protocols (Triton X-100, Trypsin, NaOH, EDTA, CHAPS, freeze–thaw + DNase, etc.). Best performing: Freeze–thaw + DNase and CHAPS retained ECM and minimized damage	HDPSCs	Cell viability, proliferation, and differentiation were significant. DSPP, DMP1 and ALP levels increased.
Elnawam et al. (2024) (18)	Bovine pulp extracellular matrix hydrogel for regenerative endodontic applications: *in vitro* characterization and *in vivo* analysis in a necrotic tooth model	Bovine pulp tissue	Trypsin + EDTA + DNase + Triton × 100; modified Bakhtiar protocol. Hydrogel prep: Pepsin/HCl digestion → neutralization → 3 mg/mL injectable hydrogel.	Rabbit DPSC	Viability was excellent and proliferation was sustained.
Smith et al. (2015) (21)	Dental pulp cell behavior in biomimetic environments	Bovine pulp	Sequential 0.5 M NaCl and 0.1 M tartaric acid (mechanical + chemical extraction) to isolate ECM proteins.	Rat and bovine dental pulp cells	Proliferation rate reduced but mineralization increased.
Song et al. (2017) (22)	Decellularized human dental pulp as a scaffold for regenerative endodontics	Human dental pulp	Compared three methods (SDS, Triton X-100 + DNase, enzymatic); one achieved maximal DNA removal <50 ng/mg and preserved ECM.	SCAPs	Good proliferation and migration and odontoblast-like cells were formed.
Bakhtiar et al. (2023) (19)	Fabrication and characterization of a novel injectable human amniotic membrane hydrogel for dentin-pulp complex regeneration	Human amniotic membrane	Decellularization: trypsin 0.25% + EDTA 0.02% 2 h at 37 °C, multiple PBS washes, stored at −80 °C. Scaffold fabrication: ECM freeze-dried → powdered, digested with pepsin + HCl, neutralized, mixed with PBS → pregel, crosslinked using genipin (0–10 mM)	HDPSC	Viability of stem cells are good in hydrogel and proliferation is good in 30 mg/mL +0.5 mm genipins
Bakhtiar et al. (2022) (23)	Human amniotic membrane extracellular matrix scaffold for dental pulp regeneration *in vitro* and *in vivo*	Human amniotic membrane	0.25% trypsin + 0.02% EDTA, washed with PBS, stored in glycerol–DMEM at –80 °C, confirmation by DNA quantification + H&E + MT staining	HDPSCs	30 gm/mL ECM showed the greatest cell migration, viability, proliferation, and adhesion

An interesting observation is the use of a wide variety of decellularization procedures, ranging from SDS treatment (up to 10%) to milder procedures such as freeze–thaw cycles, 3-[(3-cholamidopropyl) dimethylammonio]-1-propanesulfonate (CHAPS), trypsin-EDTA, and low concentration sodium dodecyl sulfate (SDS) (0.03%–0.2%). One study employing gentler detergents and enzymatic support revealed better preservation of the ECM proteins and architecture; however, the higher SDS concentrations carried the danger of upsetting the ECM. These results demonstrate that for decellularization to be successful, appropriate DNA removal and the preservation of structural and biochemical cues essential to cell activity must be balanced.

The benefit of using a dECM scaffold is backed by the number of studies that offered *in vivo* validation along with *in vitro* approval.

Hu et al. (15) demonstrated the ability of the scaffold to help in tissue regeneration when a DPSC dECM was implanted into 8-week-old mice. Shi et al. (16) found that the ability of submandibular gland-dECM, when implanted into immunodeficient mice for 12 weeks, produced pulp-like tissue with areas of high vascularity and areas of mineralization, which proved the scaffold’s excellent angiogenic and odontogenic potential.

According to Hu et al. (17), a periapical lesion-derived dECM produced pulp-like tissues *in vivo*, displaying angiogenic and odontogenic markers and promoting stem cell proliferation *in vitro*.

In a necrotic tooth model by Elnawam et al. (18), a bovine-dECM hydrogel induced a decreased inflammatory response and pulp-like tissue was developed, indicating host integration.

Bakhtiar et al. (19) supported the viability of injectable dECM hydrogels, finding that the implantation of genipin-crosslinked amniotic membrane hydrogels produced vascularized pulp-like tissue with minimal immunological reactions.

### Effect of the scaffold source and biochemical specificity

Scaffold origin emerged as an important moderator of biological performance. Dental pulp-derived dECMs consistently promoted odontoblast-like differentiation and upregulation of odontogenic markers [dentin sialophosphoprotein (DSPP), dentin matrix protein 1 (DMP1), and alkaline phosphatase (ALP)], supporting the concept of tissue-specific instructive signaling. A periapical lesion–derived dECM demonstrated pronounced angiogenic and immunomodulatory responses, including increased expression of vascular endothelial growth factor (VEGF), transforming growth factor beta (TGF-β), and fibroblast growth factor 2 (FGF-2), suggesting that inflammatory microenvironments may contribute unique proregenerative cues. By contrast, non-dental sources such as the amniotic membrane, bovine pulp, and submandibular gland provided broader ECM signaling and also supported odontogenic differentiation, albeit without the same level of tissue specificity. These findings highlight the potential to tailor the scaffold source depending on the desired biological outcome.

### Influence of decellularization protocols

The decellularization protocols varied markedly across the studies, ranging from harsh detergent-based methods (e.g., high-concentration SDS) to milder approaches employing freeze–thaw cycles, trypsin–EDTA, or low-dose detergents. Harsh protocols ensured effective DNA removal but risked disrupting the ECM ultrastructure and denaturing bioactive proteins, whereas gentler techniques preserved the ECM architecture at the expense of complete decellularization. This methodological variation underscores a persistent challenge in balancing immunological safety with biomolecular preservation and may contribute to heterogeneity in downstream stem cell responses. The emergence of injectable dECM hydrogels further expands clinical applicability by enabling minimally invasive delivery and improved canal conformity.

### Stem cell responses

DPSCs, SCAPs, and human dental pulp stem cells (HDPSCs) consistently demonstrated the following:

- high vitality and proliferation in dECM scaffolds;
- odontogenic markers, such as DSPP, DMP1, and ALP, were expressed, which proved the odontogenic differentiation properties; and
- immunomodulatory and angiogenic reactions, especially in dECMs originating from periapical lesions.

These results demonstrate dECMs' capacity to promote cell survival and lineage-specific differentiation, which is necessary for the regeneration of the dentin-pulp complex.

### Translational implications

The following in vivo findings are discussed solely to provide contextual insight into potential translational relevance and were not included as part of the systematic review evidence base.

Although the primary evidence synthesized in this review is limited to *in vitro* studies, a small number of published *in vivo* investigations have reported exploratory observations following the implantation of dECM-based scaffolds. These studies describe the formation of pulp-like tissues with varying degrees of vascularization and mineralization in experimental models using dental pulp-derived, amniotic-derived, submandibular gland-derived, and periapical lesion-derived dECMs. Such observations may offer preliminary biological context regarding how dECM scaffolds could behave *in vivo*, particularly in relation to angiogenic and odontogenic responses.

However, these findings should be interpreted with caution. The available *in vivo* studies were not systematically assessed in this review and represent early-stage exploratory models with substantial methodological variability. Importantly, no studies have evaluated long-term integration, immune response, innervation, or functional performance within the root canal system. Therefore, these *in vivo* observations do not constitute confirmatory evidence and should not be interpreted as validation of clinical efficacy. Overall, the current evidence remains pretranslational, and the conclusions of this review are based exclusively on *in vitro* data.

### Limitations of the evidence base

The findings of this review must be interpreted in light of notable limitations. First, only 10 eligible studies were identified, with no new publications after 2024, indicating both a small evidence base and a potential plateau in research activity. Several factors may explain this limited output, including technical challenges associated with reproducible dECM preparation from dental tissues, increased research emphasis on alternative biomaterials (e.g., synthetic hydrogels, peptide scaffolds, and bioactive glass), and limited translational feasibility data to justify continued exclusive *in vitro* investigations. Second, substantial methodological heterogeneity was observed in scaffold source, decellularization protocol, stem-cell type, and outcome measures, which precluded meta-analysis and reduced comparability. Third, the predominance of studies with a medium risk of bias, together with consistent absences of blinding, sample-size justification, and operator reporting, reduces confidence in the magnitude of the observed effects. Finally, the lack of clinical or late-stage preclinical studies constrains the assessment of translational potential.

### Implications and future directions

Future research should prioritize the standardization of decellularization protocols, reporting of ECM preservation metrics, and establishment of core outcome sets for dental pulp regeneration to improve reproducibility. Advanced hydrogel formats warrant further investigation due to improved deliverability and integration. Long-term *in vivo* studies evaluating vascularization, innervation, and functional endurance are necessary before early-phase clinical trials can be justified. Without such evidence, the translational trajectory of dECM scaffolds will remain speculative.

## Conclusion

This systematic review synthesized the available *in vitro* evidence on the interaction between dental-derived stem cells and decellularized extracellular matrix scaffolds within the context of regenerative endodontics. Overall, dECM scaffolds were associated with favorable cellular behaviors, including enhanced adhesion, proliferation, and expression of odontogenic and extracellular matrix-related markers. However, these observations were derived from a limited number of heterogeneous studies, the majority of which demonstrated a medium risk of bias.

Accordingly, the certainty of the current evidence remains moderate, and the findings should be interpreted as indicative rather than confirmatory. Variability in scaffold source, decellularization protocols, experimental design, and outcome reporting restricts direct comparison across studies and limits confidence in translational applicability. While dECMs represent a biologically promising scaffold strategy, their clinical relevance in regenerative dentistry cannot yet be established.

Future research should prioritize standardized decellularization methodologies, comprehensive extracellular matrix characterization, and reproducible *in vitro* models, followed by well-designed *in vivo* and early-phase clinical studies to determine their safety, functionality, and long-term regenerative outcomes.

## Data Availability

The original contributions presented in the study are included in the article/Supplementary Material, further inquiries can be directed to the corresponding author.
